# Impact of age and gender on tumor related prognosis in gastrointestinal stromal tumors (GIST)

**DOI:** 10.1186/s12885-015-1054-y

**Published:** 2015-02-14

**Authors:** Klaus Kramer, Uwe Knippschild, Benjamin Mayer, Kira Bögelspacher, Hanno Spatz, Doris Henne-Bruns, Abbas Agaimy, Matthias Schwab, Michael Schmieder

**Affiliations:** 1 Department of General and Visceral Surgery, University Hospital Ulm, Albert-Einstein-Allee 23, Ulm, 89081 Germany; 2 Institute of Epidemiology and Medical Biometry, University of Ulm, Ulm, 89075 Germany; 3 Department of General and Visceral Surgery, Krankenhaus der Barmherzigen Brüder, Romanstraße 93, München, 80639 Germany; 4 Department of Clinical, Institute of Pathology, University of Erlangen, Erlangen, 91054 Germany; 5 Dr. Margarete Fischer-Bosch Institute of Clinical Pharmacology, Stuttgart, 70376 Germany; 6 Department of Clinical Pharmacology, University Hospital, Tübingen, 72076 Germany; 7 Department of Internal Medicine, Alb-Fils-Kliniken, Goeppingen, Goeppingen, 73035 Germany; 8 Department of Anesthesiology, Alb-Fils-Kliniken, Goeppingen, Goeppingen, 73035 Germany

**Keywords:** GIST, Gastrointestinal stromal tumor, Prognosis, Outcome, Age, Gender, Sex

## Abstract

**Background:**

Risk classification and prediction of prognosis in GIST is still a matter of debate. Data on the impact of age and gender as potential confounding factors are limited. Therefore we comprehensively investigated age and gender as independent risk factors for GIST.

**Methods:**

Two independent patient cohorts (cohort I, n = 87 [<50 years]; cohort II, n = 125 [≥50 years]) were extracted from the multicentre Ulmer GIST registry including a total of 659 GIST patients retrospectively collected in 18 collaborative German oncological centers. Based on demographic and clinicopathological parameters and a median follow-up time of 4.3 years (range 0.56; 21.33) disease-specific-survival (DSS), disease-free-survival (DFS) and overall survival (OS) were calculated.

**Results:**

GIST patients older than fifty years showed significantly worse DSS compared to younger patients (p = 0.021; HR = 0.307, 95% CI [0.113; 0.834]). DSS was significantly more favorable in younger female GIST patients compared with elderly females (p = 0.008). Female gender resulted again in better prognosis in younger patients (p = 0.033).

**Conclusions:**

Patient age (<50 years) and female gender were significantly associated with a more favourable prognosis in GIST. Extended studies are warranted to confirm our clinical results and to elucidate underlying pathophysiological mechanisms.

**Electronic supplementary material:**

The online version of this article (doi:10.1186/s12885-015-1054-y) contains supplementary material, which is available to authorized users.

## Background

Based on the molecular pathogenesis of driver gain-of-function mutations in c-kit (80-90%) [1-4] and less frequently in the PDGFRα gene (5-10%), gastrointestinal stromal tumors (GIST) became a molecular model tumor in oncology emphasized by the central role of receptor tyrosine kinases in their molecular pathogenesis and the availability of small molecule inhibitor therapy.

GIST occur with an annual incidence of 7 to 20 per million [5-9]. Most patients with GIST are diagnosed within the 7^th^ decade [10,11]. Less than 10% of patients with GIST are younger than forty. There are also some single reports on pediatric GIST, which appear to be a different disease entity [12-14]. Although large-scale multi-centre studies are available (e.g. the population-based study from Sweden [5], the Surveillance, Epidemiology and End Results (SEER) database [10] and the AFIP studies [15,16], data are limited on the impact of age and gender related to risk classifications and/or prediction of prognosis in GIST. In particular it is still unclear whether prognosis of GIST in adult patients may be significantly altered by age (i.e. patients with an age younger than 50 years) and/or gender-related factors. Therefore, the aim of the present retrospective analysis was to elucidate comprehensively clinicopathological features of GIST patients younger than 50 years to identify potential age and gender-related effects on patient outcome.

## Methods

Data of the independent multicentre Ulmer GIST registry were used to extract age-dependent patient cohorts (under and above 50 years of age at diagnosis) for further comparative analyses. Patient data of the multicentre GIST registry were retrospectively obtained from 18 collaborative oncological centres in Southern-Germany between 2004 and 2009. Substantial demographic and/or social selection bias of patients could be excluded since all contributing centres are part of general or university hospitals. As previously outlined in detail [17], data registration of the multicentre Ulmer GIST registry is strictly based on clearly defined methodological criteria, such as **S**trengthening of the **R**eporting of **Ob**servational Studies in **E**pidemiology (**STROBE**) Statement and the User’s Guide to Registries Evaluating Patient Outcomes [18-21].

Briefly, all patients from study centres with proven diagnosis of GIST were consecutively included unless they refused consent. The study was approved by the Ethics Committee of the Medical Faculty of the University of Ulm (No. 90 + 91/2006). Diagnosis of GIST was based on currently applied diagnostic criteria [16,22] using histological characteristics (e.g. highly cellular spindle/epithelioid/mixed cell tumors), immunohistochemical status (positivity for KIT or PDGFRα) and mutational analysis of relevant c-kit and PDGFRα exons. Clinical data were retrospectively reviewed based on the hospital records including medical history and clinical follow-up. In addition, personal contact as well as telephone interview and/or review of medical charts in case of re-admission of patients served for data acquisition. The following parameters were defined as the most relevant clinical and clinicopathological features for the present work: age, gender, tumor localization (stomach vs. small intestine), histological subtype (spindle cell tumors vs. epithelioid/mixed cell tumors), primary tumor size (cut-off 1, 5 and 10 cm), mitotic rate (cut-off 5 and 10 per 50 HPF), immunohistochemical status of KIT or/and PDGFRα (if uncertain: mutational status), secondary malignancy (yes vs. no), risk classification according to Fletcher et al. [23] (i.e. high vs. non-high) and according to Miettinen et al. [15] (i.e. high vs. non-high), tumor recurrence and/or metastasis.

**Figure 1:**
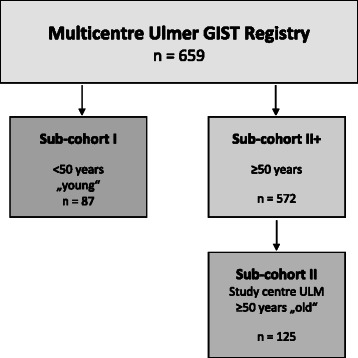
**Schematic diagram for study populations.**

At the time of data analysis for the present study, the multicentre Ulmer GIST registry consisted of 659 GIST patients (Figure 1). Since a previous clinical study by Cao et al. [24] suggested an age of 50 years as significant cut-off for the discrimination between GIST patients with worse and good prognosis, we stratified patients from our Ulmer GIST registry accordingly. 87 of the 659 GIST patients (13.2%) were younger than 50 years and defined as sub-cohort I, *“young”*. To establish a control cohort with an age of ≥50 years at time of diagnosis, all remaining 572 GIST patients of the Ulmer GIST registry older than 50 years were defined as sub-cohort II+. To ensure highest completeness of clinical and follow up data, we extracted a sub-cohort from the sub-cohort II+ that included only those GIST patients that derived from the oncology center at the University Hospital of Ulm, finally encompassing a total of 125 GIST patients (study cohort II, “old”). The overall median follow-up time for both study groups, the sub-cohort I (“young”) and sub-cohort II (“old”), was 4.3 years (range 0.56; 21.33).

### Statistical analyses

Two-sided *χ*^2^-test or Fisher’s exact test were applied, as appropriate, to check for differences of the demographic, clinical and clinicopathological parameters between the independent study-cohorts. Estimates for disease-free-survival (DFS), disease-specific-survival (DSS) and overall-survival (OS) were obtained by the Kaplan-Meier method and differences between Kaplan-Meier curves were investigated by the log-rank test. For analysis of DSS non GIST-related deaths were censored.

To prove the most relevant findings of the Kaplan-Meier analyses, an additional multivariate Cox proportional hazards regression model has been established for DSS and DFS. The variables gender, age, tumor localization have been defined as the most relevant independent variables of the model. If applicable, the Hazard Ratio (HR) and 95% confidence interval (95% CI) were calculated regarding tumor-related death and tumor recurrence and/or metastasis by applying univariate Cox proportional hazards regression models. To exclude confounding of analyses by treatment of GIST patients with the tyrosine kinase inhibitor imatinib, Kaplan-Meier analyses were recalculated, censoring all end-points and follow-ups after initiating of imatinib.

Statistical analysis was performed using SPSS V19.0 (SPSS Inc., USA). Level of significance was set to α = 0.05. Since all results rely on testing retrospective data, interpretation of hypotheses was done in an explorative manner. Therefore an adjustment of the significance level due to multiple testing has been not performed.

## Results

Table 1 comprises all demographic and clinicopathological data of GIST patients enrolled in sub-cohort I (“young”, <50 years) and sub-cohort II (“old”, ≥50 years). Whereas the gender ratio, the tumor localization and histotypes, tumor size, mitotic rate, and risk scores according to Fletcher et al. [23] and Miettinen et al. [15] were similar between both subgroups, some parameters differed age-dependently (Table 2). In patients older than 50 years small GIST tumors (<1 cm) (p = 0.002 (Fisher’s exact), OR = 11.2, 95%CI: 1.5, 87.0) as well as secondary malignancies were more frequent (p < 0.001 (*χ*^2^-Test), OR = 3.5 95% CI: 1.6, 7.2) and more GIST-related deaths occurred (p = 0.017 (Fisher’s exact), OR = 3.1, 95% CI: 1.1, 8.7). Syndromic diseases (Neurofibromatosis type 1, Carney triad) were found in three and four patients of sub-cohort I and II (both 3.4%), respectively.

**Table 1:** **Demographic and clinical data of GIST patients of sub-cohort I (<50 years,** ***“young”*** **) and sub-cohort II (≥50 years,** ***“old”*** **)**

	Parameter	Sub-cohort I (n = 87)		Sub-cohort II (n = 125)	
		<50 yr*(“young”)*		≥50 yr*(“old”)*	
**Age**					
	median (range, yr)	41.7 (14.9;49.9)		68.2 (50.9; 94.1)	
**Sex**		**n**	**%**	**n**	**%**
	female	48	55.2	68	54.4
	male	39	44.8	57	45.6
**Localization**					
	stomach	43	50.6	79	64.2
	small intestine	29	34.1	35	28.5
	colorectum	5	5.9	2	1.6
	esophagus	1	1.2	1	0.8
	EGIST	3	3.5	3	2.4
	n.d.	4	4.7	3	2.4
**Tumor size**					
	median (range, cm)	5.5 (1.2; 27.0)		4.5 (0.4;40.0)	
**Risk according to Fletcher et al.** [23]		**n**	**%**	**n**	**%**
	high	29	41.4	35	31.8
	intermediate	15	21.4	25	22.7
	low	17	24.3	31	28.2
	very Low	9	12.9	19	17.3
**Risk according to Miettinen et al.** [11]					
	high	22	33.3	30	29.7
	intermediate	10	15.2	7	6.9
	low	25	37.9	43	42.6
	very Low	9	13.6	21	20.8
**Histological subtype**					
	spindle cell	63	85.1	98	89.1
	Epithelioid/mixed	11	14.9	12	10.9
**Immunohistochemistry**					
	KIT pos	74	94.9	115	98.3
	KIT neg	4	5.1	2	1.7
	CD34 pos	48	82.8	84	84.0
	CD34 neg	10	17.2	16	16.0
	S100 pos	11	25.6	1	1.4
	S100 neg	32	74.4	68	98.6
**Clinical data**					
	Metastasis atdiagnosis	9	10.3	16	12.8
	Secondneoplasia	11	15.5	45	38.8
	R_0_resection	81	93.1	112	89.6
	Tumor debulking	4	4.6	7	7.2
	Imatinib use	24	27.6	27	21.6
**Recurrenceof disease and/ormetastasis**					
	yes	21	25.9	32	29.6
**Follow up time**					
	mean (yr, ±SD)	4.90 (3.39)		5.65 (4.55)	
	median (range, yr)	4.28 (0.59;16.31)		4.57 (0.56;21.33)	
	deceased	9	10.3	40	32.0
	alive	78	89.7	85	68.0
	tumor-relateddeath	5	5.7	20	16.0
**Survival rate**		**% (n)**		**% (n)**	
DSS (yr1/yr3/yr5)		98.5 (64)/96.6 (49)/96.6 (34)		96.2 (93)/87.0 (67)/81.2 (44)	
DFS (yr1/yr3/yr5)		88.4 (57)/81.2 (41)/78.8 (29)		79.0 (74)/74.2 (55)/69.9 (36)	
OS (yr1/yr3/yr5)		98.5 (64)/93.2 (49)/91.2 (34)		90.8 (93)/77.4 (67)/67.0 (44)	
**Syndromal disease**		3xNF1	3.4%	3x NF1	2.4%
				1x Carney	1%

yr, year; n.d., not defined; SD, standard deviation; DSS, disease specific survival; DSF, disease free survival; OS, overall survival; NF1, neurofibromatosis type 1; Carney, Carney triad (coexistence of GIST, paraganglioma and pulmonal chondroma).

**Table 2:** **Comparsion of demographic and clinicopathological parameters in sub-cohort I (** ***“young”*** **, n = 87) versus sub-cohort II (** ***“old”*** **, n = 125)**

Parameters		n	p-value*
age at diagnosis	<50 yr vs. >50 yr	212	**<0.001**
Sex	male vs. female	212	0.912
Tumor localization	stomach vs. small intestine	187	0.210
GIST histotype	spindle vs. epitheliod/mixed	184	0.426
Tumor size	<1 cm vs. ≥1 cm	199	**0.002**
	<5 cm vs. ≥5 cm	199	0.524
	<10 cm vs. ≥10 cm	199	0.605
Mitotic rate	<5 vs. ≥5 / HPF	174	0.902
	<10 vs. ≥10 / HPF	173	0.982
Risk acc.to Fletcher et al.	high vs. non-high	180	0.189
Risk acc. to Miettinen et al.	high vs. non-high	167	0.620
R_0_resection	yes vs. no	193	0.321
TKI use (imatinib)	yes vs. no	212	0.316
Secondary malignancies	yes vs. no	187	**<0.001**
Cancer related death	yes vs. no	212	**0.017**

yr, year; HPF, high power field; TKI, tyrosine kinase inhibitor;

*Two-sided *χ*^2^-test or Fisher’s exact test were applied as appropriate to check for differences between both study-cohorts.

### Survival analysis

**Figure 2:**
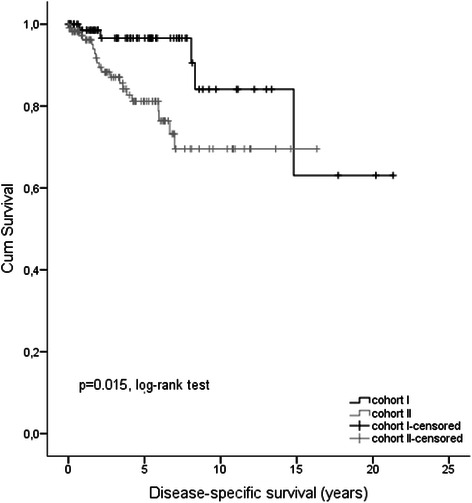
**Kaplan–Meier curves of disease-specific survival (DSS) for GIST patients of study cohort I (<50 years at diagnosis, n = 87)** ***versus*** **study cohort II (≥50 years at diagnosis, n = 125).**

At date of diagnosis the rate of metastasis was not different between sub-cohort I (10.3%) and sub-cohort II (12.8%; p = 0.586, Table 1). The outcome of GIST patients was generally more favourable in young patients (cohort I) vs. older patients (cohort II). DSS rates after 1-, 3- and 5-year follow-up in “young” vs. “old” patients were 98.5% vs. 96.2%, 96.6% vs. 87.0% and 96.6% vs. 81.2%, respectively. After 5-year follow up DSS was significantly better in GIST patients younger than 50 years (p = 0.015, log-rank-test; Figure 2). A multivariate Cox regression model adjusted for gender and tumor localization confirmed improved outcome for younger patients (p = 0.036, HR = 0.27, 95% CI: 0.079, 0.921). Moreover, we elucidated whether age as a continuous variable is an independent prognostic factor. Again we could show that the older age was associated with an increased risk for DSS (p = 0.002, HR = 1.049, 95% CI: 1.018, 1.080) and OS (p < 0.0001; HR = 1.051, 95% CI: 1.029, 1.074).

**Figure 3:**
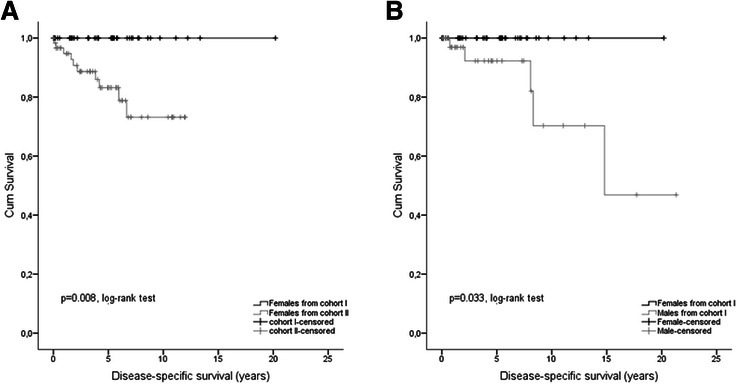
**Age and gender related outcome regarding DSS. (A)** Kaplan–Meier curves of disease-specific survival (DSS) for female GIST patients of study cohort I (<50 years at diagnosis) *versus* study cohort II (≥50 years at diagnosis). **(B)** Kaplan–Meier curves of disease-specific survival (DSS) for gender-related differences of GIST patients younger than 50 years at diagnosis (study cohort I).

Next we investigated differences for DSS rates between “young” and “old” GIST patients (sub-cohort I vs. II) considering selected demographic and clinicopathological parameters as well as different risk scores as given in Table 3 and Additional file 1: Table S1. Most strikingly a more favourable DSS after 5 years was found in female “young” patients (p = 0.008, log-rank-test, Figure 3A), but not in men. Calculation of the corresponding HR failed since only censored events were observed in sub-cohort I. Moreover, DSS was better for “young” GIST patients with high risk classification according to Fletcher et al. [23] (p = 0.004;HR = 0.15, 95% CI: 0.04; 0.66), tumor size above 5 cm (p = 0.008; HR = 0.11, 95% CI: 0.01; 0.81), a mitotic rate ≥5/50 HPF (p = 0.026; HR = 0.22, 95% CI: 0.05; 0.95) and tumor localization in the stomach (p = 0.036; HR = 0.15, 95% CI:0.02; 1.17) according to univariate Cox regression models.

**Table 3:** **Disease-specific survival (DSS) for GIST patients <50 years (sub-cohort I,** ***“young”*** **) versus ≥50 years (sub-cohort II,** ***“old”*** **) related to GIST relevant clinicopathological parameters**

Parameter		Disease-specific survival (DSS) rates						p-value^1^
		Sub-cohort I (*“young”*)			Sub-cohort II (*“old”*)			
		n = 87			n = 125			
		1 yr	3 yr	5 yr	1 yr	3 yr	5 yr	
Sex	male	96.9%	92,3%	92,3%	97,8%	84,8%	78,5%	0.326
	female	100%	100%	100%	94,7%	88,6%	83,2%	**0.008**
	**p-value** ^2^		**0.033**			0.596		
Localization	Gaster	97,2%	97,2%	97,2%	97,0%	88,0%	83,4%	**0.036**
	Small intestine	100%	93,8%	93,8%	96,4%	88,5%	78,6%	0.267
	**p-value** ^2^		0.225			0.813		
Histotype	Spindle	100%	97,4%	97,4%	96,4%	90,8%	83,5%	**0.028**
	epitheliod/mixed	100%	100%	100%	91,7%	66,7%	66,7%	0.061
	**p-value** ^2^		0.695			0.097		
Size	<1 cm	-	-	-	100,0%	100,0%	85,7%	-
	≥1 cm	100%	97,9%	97,9%	96,6%	87,5%	82,6%	**0.012**
	**p-value** ^2^		-			0.499		
Size	<5 cm	96,8%	92,2%	92,2%	100,0%	94,5%	90,9%	0.630
	≥5 cm	100%	100%	100%	94,1%	83,8%	76,3%	**0.008**
	**p-value** ^2^		0.462			**0.012**		
Size	<10 cm	97,9%	95,0%	95,0%	98,6%	95,3%	93,1%	0.839
	≥10 cm	100%	100%	100%	92,0%	71,1%	56,0%	**0.010**
	**p-value** ^2^		0.759			**<0.001**		
Mitotic rate	<5 / 50 HPF	100%	100%	100%	98,4%	96,2%	90,4%	0.131
	≥5 / 50 HPF	100%	100%	100%	91,6%	73,7%	66,7%	**0.026**
	**p-value** ^2^		**0.038**			**0.001**		
Mitotic rate	<10 / 50 HPF	100%	100%	100%	97,4%	92,4%	88,2%	**0.043**
	≥10 / 50 HPF	100%	100%	100%	89,7%	68,6%	56,1%	**0.025**
	**p-value** ^2^		**<0.001**			**<0.001**		
Risk (NIH)	high	100%	100%	100%	87,9%	68,4%	60,8%	**0.004**
	non-high	100%	100%	100%	100,0%	98,0%	92,9%	0.227
	**p-value**		**0.027**			**<0.001**		
Risk (AFIP)	high	100%	100%	100%	89,4%	70,5%	61,7%	**0.018**
	non-high	100%	100%	100%	98,5%	94,4%	89,4%	0.084
	**p-value**		**0.013**			**<0.001**		

^1^Unadjustedp-values comparing data from study-cohort I vs. II considering DSS after 5 year follow-up.

^2^Unadjusted p-values comparing data within study-cohort I and II considering DSS rates after 5 year follow-up.

Additional analyses regarding DSS after 5 years in relationship to demographic and clinicopathological parameters as well as different risk scores in each sub-cohort revealed a more favourable outcome in “young” female patients (p = 0.033,log-rank test; Table 3 and Additional file 1: Table S2, Figure 3B) whereas DSS was not gender-specific different (p = 0.596) in sub-cohort II (“old"). Moreover DSS was improved in “young” patients with non-high risk GIST (p = 0.027) and with tumors characterized by a mitotic rate below 5/50 HPF (p = 0.038). DSS was also significantly improved in “old” patients with non-high risk GIST (p < 0.001, HR = 0.09, 95% CI: 0.03; 0.31), in GIST with mitotic rate <10/50HPF (p < 0.001, HR = 0.15, 95% CI: 0.06; 0.39) and with tumors sized <5 cm (p = 0.012, HR = 0.23, 95% CI: 0.07; 0.81).

DFS-rates for the follow-up times of 1-, 3-, and 5-years were 88.4%, 81.2% and 78.8% in sub-cohort I as compared to 79.0%, 74.2% and 69.6% in sub-cohort II (Table 1), indicating no significant differences (p = 0.364, log-rank-test; p = 0.916, multivariate Cox model adjusted for gender and tumor localization; HR = 0.968, 95% CI: 0.534, 1.756). Regarding tumor size ≥10 cm (p = 0.014, HR = 0.36, 95% CI: 0.14;0.90), mitotic rate ≥10/50 HPF (p = 0.011; HR = 0.34, 95% CI: 0.12; 0.92) and high-risk classification (p = 0.011; HR = 0.44, 95% CI: 0.22; 0.89) DFS was more favourable in “young” GIST patients (detailed data regarding log-rank test and OR at five years see Additional file 1: Table S3).

**Figure 4:**
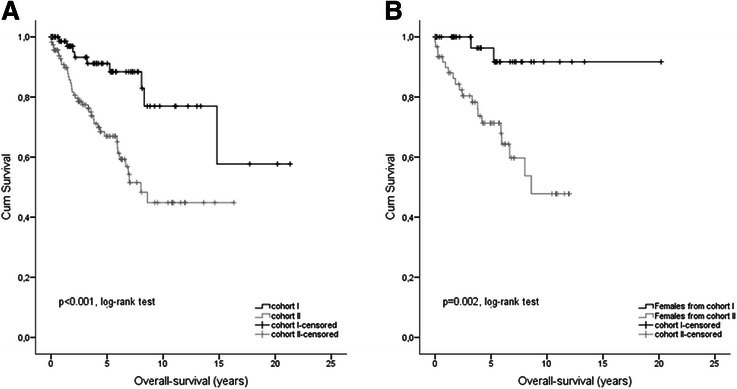
**Age and gender related outcome regarding OS. (A)** Kaplan–Meier curves of overall survival (OS) for GIST patients of study cohort I (<50 years at diagnosis) *versus* study cohort II (≥50 years at diagnosis). **(B)** Kaplan–Meier curves of overall survival (OS) for female GIST patients of study cohort I (<50 years at diagnosis) *versus* study cohort II (≥50 years at diagnosis).

OS-rates were compared after 1-, 3- and 5-year follow up between sub-cohort I (98.5%, 93.2% and 91.2%) and sub-cohort II (90.8%, 77.4% and 67.0%, Table 1). Again GIST patients younger than 50 years showed a more favourable outcome which was significantly different (p <0.001; HR = 0.292, 95% CI: 0.140; 0.606, Figure 4A). Regarding gender aspects again female patients particularly with an age <50 years showed better OS (p = 0.002, log-rank test; p = 0.008, cox model; HR = 0.141, 95% CI: 0.033; 0.604, Figure 4B).

**Figure 5:**
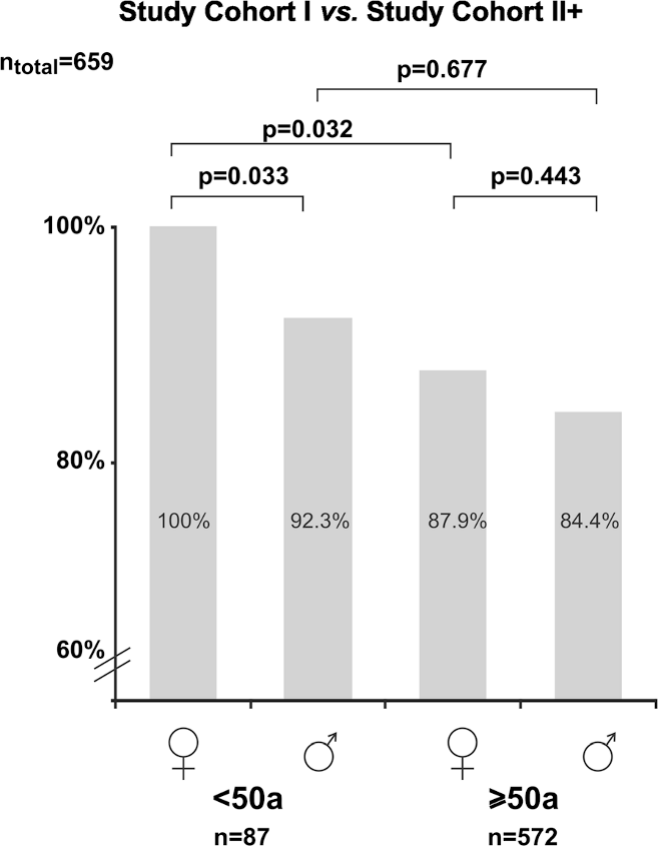
**Summary of unadjusted** ***p*** **-values for disease-specific survival (DSS) of male and female GIST patients of study cohort I (<50 years at diagnosis, n = 87)** ***versus*** **study cohort II+ (≥50 years at diagnosis, n = 572) after 5 year follow-up.**

To replicate the association of clinical outcome data regarding age and gender we used study cohort II+ which included 572 GIST patients of the Ulmer GIST registry older than 50 years (Figure 1). The descriptive and clinical data if provided for cohort II+ (Additional file 1: Table S4) were not different compared to cohort II “old” (Table 1). However, the median follow-up time of cohort II+ was 3.25 years (range 0.01; 21.33) and approximately one year shorter compared with cohort II (4.57 years, range 0.56; 21.33). As shown by Figure 5 more favourable outcome was found again for young female GIST patients (<50 years) comparing DSS-rates after a 5 year follow-up (p = 0.032, log-rank test).

## Discussion

The frequencies of GIST in men (54%) and women (46%) [6] are quite similar. About three quarters of GIST are diagnosed in patients aged above 50 years (median 58 years [25]). In population based series including cases diagnosed at autopsy, the median age was approximately ten years older (66 to 69 years) [5,7]. Combined data on age and gender related to clinicopathological findings of GIST and/or prognosis are limited. This may be of importance since gender-related effects (e.g. hormonal status) in younger GIST patients may contribute to GIST prognosis.

Here, we present an observational study, evaluating comprehensively clinicopathological features of GIST and patient outcome to elucidate more deeply the role of patient’s age and gender on the prognosis of GIST. We analyzed 87 GIST patients younger than fifty years (sub-cohort I) and compared these study cohort with data from a single-center collective of patients older than 50 years (n = 125, sub-cohort II). Both collectives are part of the multicentre Ulmer GIST registry, encompassing a total of 659 GIST patients at the time of study evaluation.

First, our data demonstrate that generally the distribution of gender, tumor localization, histotype, KIT status, mitotic rate, median tumor size and risk classification by different risk scores are similar between patients younger or older than 50 years at time of diagnosis, in concordance with data of large series of GIST patients [10,15]. More detailed analyses however revealed a significant higher occurrence of small sized GIST (<1 cm) in patients ≥50 years (sub-cohort II, p = 0.002, OR = 11.2, 95%CI: 1.5; 87.0, Table 2). This might be explained by the fact that diagnostic (e.g. endoscopies, radiological scans etc.) as well as surgical procedures are more frequently performed in this age group with a higher frequency of GIST diagnoses as an incidental finding. Autopsy data also support this assumption indicating that 10 to 35% of histologically investigated stomach tissues contain GIST-tumorlets (micro-GIST [26-28]. As expected, elderly patients (sub-cohort II) showed a significantly higher percentage of secondary malignancies (38.8% vs. 15,5%, p < 0.001, OR = 3.5 [1.6; 7.2]). Generally, the occurrence of secondary neoplasia in both GIST cohorts (sub-cohort I plus II) with 29.9% are comparable to published data, reporting secondary malignancies between 14% and 42% of GIST patients [29-31].

The first most striking result of our study is a significantly more favorable DSS rate after 5 year follow up for patients younger than 50 years in comparison to older patients (p = 0.015, log-rank-test; Figure 2) although patients ≥50 years showed significantly more often smaller tumors (<1 cm). The beneficial prognostic effect held true for OS (p < 0.001, log-rank-test; Figure 4B) in younger patients but was not seen regarding DFS (p = 0.364). Our data are supported by Tran et al. [6] who reported that older age (>65 years) was an independent predictor of mortality (OS) in GIST patients. In contrast a study including 188 patients showed that younger age (<50 yrs) was associated with worse prognosis in GIST (p = 0.035), highlighting a putative beneficial prognostic value of older age in GIST [24]. Reasons for this discrepancy may be due to the limited number of patients in the study by Cao et al. as well as the clinical endpoint OS used by the authors. Since, about 50% of death in GIST patients are not GIST-related, supported by our data, DSS may be a more appropriate clinical endpoint in GIST for outcome analyses.

The second interesting result of our study was a gender-related difference in patient outcome. Only younger women showed better DSS (p = 0.008, Figure 3A) and this effect held true after comparison of young female vs. male GIST patients in cohort I (p = 0.033, Figure 3B). To exclude confounding by the use of the tyrosine kinase inhibitor Imatinib, Kaplan-Meier analyses for DSS were recalculated by censoring all patients who received TKI treatment, resulting again in a more favourable prognosis of young females (p = 0.047). These results are in accordance with data from Miettinen et al. who reported an excellent long-term-prognosis particularly in female patients younger than 21 years and gastric GIST [13]. In addition, male gender was associated by some authors with a more worse outcome [32,33].

The underlying mechanism for the gender-related more favorable prognosis of GIST in patients younger than <50 years remains unclear. There may be a relationship to the reproductive age in younger females or to the use of contraceptive medication but this is speculative and several confounding factors need to be considered.

Young females are significantly overrepresented among gastric GIST patients aged <40 years (>80%) [34-38]. Current knowledge confirms that the majority of GIST in young adults as well as in children, particularly female patients, representing a distinctive disease entity different from the kinase mutated GIST in adults (so-called type 1 GIST). This subtype of GIST harbors molecular alterations in the mitochondrial enzymatic cascade succinate dehydrogenase (SDH). Mutations in any of the four SDH subunits (A,B,C,D), either germline or somatic, result in complete loss of the nuclear expression of the subunit B shown by results of immunohistochemistry (SDHB-deficient or type 2 GIST) [34-41]. Patients with germline mutations in SDHB may develop both GIST and paraganglioma (= Carney-Stratakis syndrome) [42]. On the other hand, patients with the non-hereditary Carney triad (GIST, pulmonary chondroma and paraganglioma) lack mutations in the SDH complex. Instead, epigenetic silencing of the SDH subunit C by DNA methylation as a novel non-heritable mechanism for the development of Carney triad-associated GIST may be more important [43]. Common to the heterogeneous type 2 GISTs are the early age of onset of disease before 40 years and a striking female predilection of >80% except SDH subunit A mutated cases which occur at relatively higher age and affect both genders. Thus, regarding the prognostic impact of age and gender, some of the young females of our study cohort might have had type 2 GIST. Nevertheless, given the low prevalence of SDHB-deficient GIST of about 7% among gastric GIST [36], it appears to be unlikely that a predominance of type 2 GIST may explain entirely the age group effect of our study.

## Conclusions

In summary, we present first data on the prognostic impact of age and gender in patients with GIST. The favourable outcome in the young age group which is gender-specific remains currently poorly understood. The real impact of age- and gender-related biological and pathophysiological factors on the prognosis in GIST warrants further prospective studies on larger cohorts with matched genotype and tumor site.

## Additional file

Additional file 1: Table S1. Intercollective analysis: Summary of Kaplan-Meier-analyses for disease-specific survival (DSS) and disease-free survival (DFS) after 5 year follow up of 5 years in GIST patients of study-cohort I (< 50 year) *versus* study-cohort II (≥50 year).0020. **Table S2.** Intracollective analysis: Summary of Kaplan-Meier-analyses for disease-specific survival (DSS) and disease-free survival (DFS) after 5 year follow up in GIST patients within study-cohort I (<50 years) and study cohort II (≥50 year). **Table S3.** Disease-free survival (DFS) for GIST patients <50 years (sub-cohort I *“young”*) versus ≥50 years (sub-cohort II *“old”*) related to GIST relevant clinicopathological parameters.

## Abbreviations

CI: Confidence interval
DFS: Disease-free-survival
DSS: Disease-specific-survival
E.g.: “Exempli gratia” (for example)
GIST: Gastrointestinal stromal tumor
HR: Hazard Ratio
HPF: High power field
I.e.: Id est
NF: Neurofibromatosis
OR: Odds ratio
PDGFRα: Platlet-derived growth factor alpha
SD: Standard deviation
SEER: Surveillance Epidemiology and End Results
STROBE: **S**trengthening of the **R**eporting of **O**bservational **S**tudies in **E**pidemiology
TKI: Tyrosine kinase inhibitor
Yr: Year(s)
