# Supplementary material for: Impact of age and gender on tumor related prognosis in gastrointestinal stromal tumors (GIST)
Source: BMC Cancer. 2015 Feb 14;15:57. doi: 10.1186/s12885-015-1054-y (PMC4384379; doi:10.1186/s12885-015-1054-y)
Supplement: Additional file 1: Table S1. — Intercollective analysis: Summary of Kaplan-Meier-analyses for disease-specific survival (DSS) and disease-free survival (DFS) after 5 year follow up of 5 years in GIST patients of study-cohort I (< 50 year) versus study-cohort II (≥50 year).0020. Table S2. Intracollective analysis: Summary of Kaplan-Meier-analyses for disease-specific survival (DSS) and disease-free survival (DFS) after 5 year follow up in GIST patients within study-cohort I (<50 years) and study cohort II (≥50 year). Table S3. Disease-free survival (DFS) for GIST patients <50 years (sub-cohort I “young”) versus ≥50 years (sub-cohort II “old”) related to GIST relevant clinicopathological parameters. [file 12885_2015_1054_MOESM1_ESM.doc]

**Additional file 1**

**Table S1**

**Intercollective analysis: Summary of Kaplan-Meier-analyses for disease-specific survival (DSS) and disease-free survival (DFS) after 5 year follow up of 5 years in GIST patients of study-cohort I (< 50 year) *versus* study-cohort II (≥50 year)**

|  | | **Disease-specific survival** | | **Disease-free survival** | |
| --- | --- | --- | --- | --- | --- |
| **strata** | | **p-value** (log-rank-test) | **Odds ratio,**  **95% convidence interval** | **p-value** (log-rank-test) | **Odds ratio,**  **95% convidence interval** |
| sex |  |  |  |  |  |
|  | male | 0.326 | OR=2.9 [0.5; 15.9] | 0.419 | OR=1.7 [0.5; 5.6] |
|  | female | **0.008** | - | 0.613 | OR=1.4 [0.5; 3.9] |
| localization | |  |  |  |  |
|  | gaster | **0.036** | OR=5 [0.6; 42.8] | 0.759 | OR=0.9 [0.3; 2.8] |
|  | small intestine | 0.267 | OR=4.6 [0.5; 45.4] | 0.863 | OR=1.4 [0.4; 5.1] |
| histotype | |  | - |  |  |
|  | spindle | **0.028** | OR=8.2 [1; 67.3] | 0.537 | OR=2.1 [0.8; 5.4] |
|  | epitheliod/mixed | 0.051 | - | 0.492 | OR=1 [0.1; 7.1] |
| size |  |  |  |  |  |
|  | <1cm | censored |  | censored | - |
|  | ≥1cm | **0.012** | OR=9.5 [1.2; 76.7] | 0.178 | OR=1.8 [0.8; 4.3] |
|  | <5cm | 0.630 | OR=1.1 [0.2; 7.6] | 0.383 | OR=0.4 [0.1; 2.9] |
|  | ≥5cm | **0.008** | - | 0.104 | OR=1.9 [0.7; 5.4] |
|  | <10cm | 0.839 | OR=1.4 [0.2; 8] | 0.471 | OR=0.8 [0.3; 2.7] |
|  | ≥10cm | **0.010** | - | **0.014** | OR=4.8 [1.1; 20.5] |
| mitotic rate | |  |  |  |  |
|  | <5 / 50 HPF | 0.131 | - | 0.539 | OR=0.9 [0.1; 6.1] |
|  | ≥ 5 / 50 HPF | **0.026** | - | **0.033** | OR=2.4 [0.7; 7.9] |
|  | <10 / 50 HPF | **0.043** | - | 0.657 | OR=0.9 [0.2; 3.6] |
|  | ≥ 10 / 50 HPF | **0.025** | - | **0.011** | OR=9 [1.3; 61.1] |
| risk acc. to  Fletcher et al. | |  |  |  |  |
|  | High | **0.004** | - | **0.011** | OR=5.4 [1.6; 18.3] |
|  | non-high | 0.227 | - | 0.565 | OR=0.9 [0.1; 11.2] |
| risk acc. to  Miettinen et al. | |  |  |  |  |
|  | High | **0.018** | - | 0.207 | - |
|  | non-high | 0.084 | - | 0.869 | OR=2.3 [0.2; 22] |

**Table S2**

**Intracollective analysis: Summary of Kaplan-Meier-analyses for disease-specific survival (DSS) and disease-free survival (DFS) after 5 year follow up in GIST patients within study-cohort I (<50 years) and study cohort II (≥50 year)**

|  | | **Disease-specific survival** | | **Disease-free survival** | |
| --- | --- | --- | --- | --- | --- |
| **Strata** | | **p-value** (log-rank-test) | **Odds ratio,**  **95% convidence interval** | **p-value** (log-rank-test) | **Odds ratio,**  **95% convidence interval** |
| sex (male vs. female) | |  |  |  |  |
|  | collective I | **0.033** | OR=1.2 [0.9; 1.4] | 0.876 | OR=0.9 [0.2; 3] |
|  | collective II | 0.596 | OR=1.4 [0.5; 4.6] | 0.793 | OR=1.1 [0.4; 2.7] |
| localization  (gaster vs. small intestine) | |  |  |  |  |
|  | collective I | 0.225 | OR=1.3 [0.1; 23.5] | 0.284 | OR=1.3 [0.3; 4.7] |
|  | collective II | 0.813 | OR=1.2 [0.3; 4.4] | 0.493 | OR=1.9 [0.7; 5.6] |
| histotype (spindle vs.  epitheliod/mixed cell) | |  |  |  |  |
|  | collective I | 0.695 | - | 0.265 | OR=0.3 [0.1; 1.8] |
|  | collective II | 0.097 | OR=0.6 [0.1; 2.2] | 0.124 | OR=0.6 [0.2; 2.2] |
| size (<1cm vs. ≥1cm) | |  |  |  |  |
|  | collective I | censored | - | 0.728 | - |
|  | collective II | 0.499 | OR=1 [0.1; 9.9] | 0.137 | - |
| size (<5cm vs. ≥5cm) | |  |  |  |  |
|  | collective I | 0.462 | - | 0.099 | OR=3.2 [0.7; 14.3] |
|  | collective II | **0.012** | OR=3.1 [0.7; 12.5] | **<0.001** | OR=14.4 [3; 68.4] |
| size (<10cm vs. ≥10cm) | |  |  |  |  |
|  | collective I | 0.759 | - | 0.164 | OR=2.6 [0.7; 10.6] |
|  | collective II | **<0.001** | OR=8.5 [2.2; 33] | **<0.001** | OR=15.2 [4.4; 51.9] |
| mitotic rate  (<5 vs. ≥5 / 50 HPF) | |  |  |  |  |
|  | collective I | **0.038** | - | **0.004** | OR=5.6 [1; 32.5] |
|  | collective II | **0.001** | OR=3.7 [1; 13.7] | **<0.001** | OR=14.7 [3.7; 58.2] |
| mitotic rate  (<10 vs. ≥10 / 50 HPF) | |  |  |  |  |
|  | collective I | **<0.001** | - | **<0.001** | OR=5 [1; 25.8] |
|  | collective II | **<0.001** | OR=5.4 [1.5; 19.8] | **<0.001** | OR=47.6 [9; 252.5] |
| risk acc. to Fletcher et al.  (high vs. non-high) | |  |  |  |  |
|  | collective I | **0.027** | - | **0.001** | OR=10 [1.1; 91.4] |
|  | collective II | **<0.001** | OR=8 [1.9; 33] | **<0.001** | OR=57.1 [10.9; 299] |
| risk acc. toMiettinen et al.  (high vs. non-high) | |  |  |  |  |
|  | collective I | **0.013** | - | **<0.001** | OR=19.4 [2; 185.7] |
|  | collective II | **<0.001** | OR=4.5 [1.3; 16] | **<0.001** | OR=185.7 [5; 70.7] |

**Table S3**

**Disease-free survival (DFS) for GIST patients <50 years (sub-cohort I *“young”*) versus ≥50 years (sub-cohort II *“old”*) related to GIST relevant clinicopathological parameters**

| **Parameter** | |  | **disease-free survival (DFS) rates** | | | | | | | | | | **p-value^1^** |
| --- | --- | --- | --- | --- | --- | --- | --- | --- | --- | --- | --- | --- | --- |
|  | |  | Sub-Cohort I (*„young“*)  n=87 | | | | | Sub-Cohort II (*„old“*)  n=125 | | | | |  |
|  | |  | 1yr | 3yr | | | 5yr | 1yr | | 3yr | | 5yr |  |
| sex | male | | 83,6% | | 83,6% | 83,6% | | 79,4% | 76,6% | | 70,0% | | 0.419 |
|  | female | | 92,7% | | 79,7% | 75,3% | | 78,5% | 72,3% | | 69,1% | | 0.613 |
|  | **p-value**^2^ | |  | | 0.876 |  | |  | 0.793 | |  | |  |
| localization | gaster | | 89,6% | | 82,5% | 77,4% | | 81,1% | 77,2% | | 74,9% | | 0.759 |
|  | smallintestine | | 81,2% | | 69,7% | 69,7% | | 75,4% | 71,4% | | 67,2% | | 0.863 |
|  | **p-value**^2^ | |  | | 0.284 |  | |  | 0.493 | |  | |  |
| histotype | spindle | | 89,7% | | 84,9% | 84,9% | | 82,7% | 78,2% | | 72,4% | | 0.537 |
|  | epitheliod/mixed | | 90,0% | | 56,3% | 56,3% | | 58,3% | 50,0% | | 50,0% | | 0.492 |
|  | **p-value**^2^ | |  | | 0.265 |  | |  | 0.124 | |  | |  |
| size | <1cm | | - | | - | - | | 77,6% | 72,2% | | 67,2% | | - |
|  | ≥1cm | | 88,8% | | 82,7% | 82,7% | | 100,0% | 100,0% | | 100,0% | | 0.178 |
|  | **p-value**^2^ | |  | | 0.728 |  | |  | 0.137 | |  | |  |
| size | <5cm | | 91,4% | | 91,4% | 91,4% | | 96,0% | 96,0% | | 96,0% | | 0.383 |
|  | ≥5cm | | 84,0% | | 73,0% | 73,0% | | 64,2% | 55,9% | | 48,7% | | 0.104 |
|  | **p-value**^2^ | |  | | 0.099 |  | |  | **<0.001** | |  | |  |
| size | <10cm | | 88,9% | | 88,9% | 88,9% | | 92,2% | 87,4% | | 87,1% | | 0.471 |
|  | ≥10cm | | 83,3% | | 62,5% | 62,5% | | 42,9% | 38,6% | | 23,5% | | **0.014** |
|  | **p-value**^2^ | |  | |  | 0.164 | |  | **<0.001** | |  | |  |
| mitotic rate | <5 / 50 HPF | | 97,4% | | 92,9% | 92,9% | | 94,9% | 94,9% | | 94,9% | | 0.539 |
|  | ≥ 5 / 50 HPF | | 78,8% | | 68,3% | 68,3% | | 49,5% | 40,7% | | 37,8% | | **0.033** |
|  | **p-value**^2^ | |  | | **0.004** |  | |  | **<0.001** | |  | |  |
| mitotic rate | <10 / 50 HPF | | 95,9% | | 89,4% | 89,4% | | 91,8% | 90,0% | | 90,0% | | 0.657 |
|  | ≥ 10 / 50 HPF | | 67,7% | | 59,2% | 59,2% | | 28,6% | 19,0% | | - | | **0.011** |
|  | **p-value**^2^ | |  | | **<0.001** |  | |  | **<0.001** | |  | |  |
| risk (NIH) | high | | 80,6% | | 65,5% | 65,5% | | 43,5% | 34,2% | | 23,7% | | **0.011** |
|  | non-high | | 97,4% | | 97,4% | 97,4% | | 96,8% | 96,8% | | 96,8% | | 0.565 |
|  | **p-value**^2^ | |  | | **0.001** |  | |  | **<0.001** | |  | |  |
| risk (AFIP) | high | | 67,7% | | 52,6% | 52,6% | | 39,3% | 32,1% | | 28,6% | | 0.207 |
|  | non-high | | 100,0% | | 96,3% | 96,3% | | 93,4% | 93,4% | | 93,4% | | 0.869 |
|  | **p-value**^2^ | |  | | **<0.001** |  | |  | **<0.001** | |  | |  |

^1^unadjustedp-values comparing data from study-cohort I vs. II considering DFS after 5 year follow-up

^2^unadjusted p-values comparing data within study-cohort I and II considering DFS rates after 5 year follow-up

**Table S4**

**Demographic and clinical data of GIST patients of sub-cohort II+ (≥50 years)**

| **Parameter** | | **n (%)** | **Sub-Cohort II+** | |
| --- | --- | --- | --- | --- |
|  |  |  | ≥ 50yr | |
| **Age** |  | 572 (100.0) |  |  |
|  | median (range;yr) |  | 68.9 (50.3; 94.1) | |
| **Sex** | | 572 (100.0) | **n** | **%** |
|  | female |  | 285 | 49.8 |
|  | male |  | 287 | 50.2 |
| **Localization** | | 562 (98.3) |  |  |
|  | stomach |  | 374 | 66.5 |
|  | Small intestine |  | 146 | 26.0 |
|  | colorectum |  | 27 | 4.8 |
|  | esophagus |  | 2 | 0.4 |
|  | EGIST |  | 8 | 1.4 |
|  | n.d. |  | 5 | 0.9 |
| **Tumor size** | |  |  |  |
|  | median (range. cm) |  | 4.4 (0.2; 40.0) | |
| **Risk according to Fletcher et al.** | | 463 (80.1) | **n** | **%** |
|  | high |  | 117 | 25.3 |
|  | intermediate |  | 110 | 23.8 |
|  | low |  | 161 | 34.8 |
|  | very Low |  | 75 | 16.2 |
| **Risk according to Miettinen et al.** | | 439 (76.7) |  |  |
|  | high |  | 91 | 20.7 |
|  | intermediate |  | 58 | 13.2 |
|  | low |  | 201 | 45.8 |
|  | verylow |  | 89 | 20.3 |
| **Histological subtype** | | 481 (84.1) |  |  |
|  | spindle cell |  | 418 | 86.9 |
|  | Epithelioid/mixed |  | 63 | 13.1 |
| **Immunohistochemistry** | |  |  |  |
|  | KIT pos | 517 (90.4) | 501 | 96.9 |
|  | KIT neg | 517 (90.4) | 16 | 3.1 |
|  | CD34 pos | 399 (69.8) | 359 | 90.0 |
|  | CD34 neg | 399 (69.8) | 40 | 10.0 |
|  | S100 pos | 313 (54.7) | 57 | 18.2 |
|  | S100 neg | 313 (54.7) | 256 | 81.8 |
| **Clinical data** | |  |  |  |
|  | metastasis at diagnosis | 572 (100.0) | 30 | 5.2 |
|  | mecondneoplasia | 572 (100.0) | 160 | 28.0 |
|  | R_0_ resection | 458 (80.1) | 418 | 91.3 |
|  | imatinibuse | 570 (99.7) | 88 | 15.4 |
| **Recurrence of disease and/or metastasis** | |  |  |  |
|  | yes | 468 (81.8) | 101 | 21.6 |
| **Follow up time** | | 572 (100.0) |  |  |
|  | mean (y. ±SD) |  | 3.70 (3.00) | |
|  | median (range;yr) |  | 3.25 (0.01; 21.33) | |
|  | deceased |  | 132 | 23.1 |
|  | alive |  | 440 | 76.9 |
|  | tumor-relateddeath | 569 (99.5) | 53 | 9.3 |
| **Survival rate** | |  | **%** | |
| DSS (yr1 / yr3 / yr5) | | 386 / 258 / 140 | 96.7 / 91.0 / 86.1 | |
| DFS (yr1 / yr3 / yr5) | | 311 / 209 / 111 | 87.5 / 83.5 / 80.0 | |
| OS (yr1 / yr3 / yr5) | | 386 / 258 / 140 | 91.7 / 79.8 / 70.2 | |
| **Syndromal disease** | | 334 (58.4) | **n** | **%** |
|  | |  | 9 x NF1 | 2.7 |
|  |  |  | 1x Carney | 0.3 |

yr, year
